# Stress, Hyperglycemia, and Insulin Resistance Correlate With Neutrophil Activity and Impact Acute Myocardial Infarction Outcomes

**DOI:** 10.7759/cureus.63731

**Published:** 2024-07-03

**Authors:** Elena Barbu, Andreea Mihaila, Alexandru Filippi, Andra Stoenescu, Letitia Ciortan, Elena Butoi, Cristina Beiu, Marius N Popescu, Serban Balanescu

**Affiliations:** 1 Department of Cardiology, Elias Emergency University Hospital, Carol Davila University of Medicine and Pharmacy, Bucharest, ROU; 2 Department of Inflammation, Institute of Cellular Biology and Pathology Nicolae Simionescu, Bucharest, ROU; 3 Department of Biochemistry and Biophysics, Carol Davila University of Medicine and Pharmacy, Bucharest, ROU; 4 Department of Cardiology, Carol Davila University of Medicine and Pharmacy, Bucharest, ROU; 5 Department of Oncologic Dermatology, Elias Emergency University Hospital, Carol Davila University of Medicine and Pharmacy, Bucharest, ROU; 6 Department of Physical Medicine and Rehabilitation, Carol Davila University of Medicine and Pharmacy, Bucharest, ROU

**Keywords:** neutrophil-to-lymphocyte ratio, stress hyperglicemia, inflammatory markers, acute myocardial infarction, acute insulin resistance

## Abstract

Introduction

Acute insulin resistance (IR) and hyperglycemia are frequently observed during acute myocardial infarction (AMI), significantly influencing both immediate and long-term patient outcomes, irrespective of diabetic status. Neutrophilia and increased neutrophil activity, which are common in these scenarios, have been associated with poorer prognoses, as demonstrated in our recent findings. While it is well established that neutrophils and stress-induced hyperglycemia exacerbate inflammation and hinder recovery, the complex interplay between these factors and their combined impact on AMI prognosis remains inadequately understood. This study aims to investigate the effects of stress hyperglycemia and IR on AMI patients at the onset of the event and to elucidate the relationship between these metabolic disturbances and inflammatory markers, particularly neutrophils.

Methods

We conducted a longitudinal prospective study on 219 AMI patients at Elias Emergency Hospital in Bucharest, Romania, from April 2021 to September 2022. Patients were included within 24 hours of AMI with ST-segment elevation and excluded if they had acute infections or chronic inflammatory diseases. Blood samples were collected to study inflammatory biomarkers, including neutrophil extracellular traps (NETs), S100A8/A9, interleukin (IL)-1β, IL-18, and IL-6. Diabetic and pre-diabetic statuses were defined using glycated hemoglobin (HbA1c) and medical history (ADA 2019 criteria). To assess glycemic parameters, we employed the glycemia ratio (GR) and the homeostatic model assessment of insulin resistance (HOMA-IR) index, enabling a precise evaluation of stress hyperglycemia, acute IR, and their prognostic implications. Patients were stratified into groups based on GR calculations, categorized as under-average glycemia, normal glycemia, and stress hyperglycemia.

Results

The majority of patients in the stress hyperglycemia group exhibited an unfavorable prognosis. This group also demonstrated significantly elevated neutrophil counts and neutrophil-to-lymphocyte ratios (NLR). The GR was significantly and positively correlated with inflammation markers, including neutrophil count (Pearson's R = 0.181, P = 0.008) and NLR (Pearson's R = 0.318, P < 0.001), but showed no significant correlation with other evaluated inflammatory markers.

Conclusions

Our findings suggest that poor outcomes in AMI patients may be associated with stress hyperglycemia, as indicated by GR. AcuteIR, quantified by GR and HOMA-IR, exhibits a strong correlation with neutrophil count and NLR within the first 24 hours of AMI onset. However, no significant correlation was observed with other inflammatory markers, such as IL-1β, IL-18, and IL-6, underscoring the specific interplay between IR and neutrophil activity in this setting.

## Introduction

Acute insulin resistance (IR) and hyperglycemia significantly impact the pathophysiology of acute myocardial infarction (AMI), contributing to its initiation and progression. It affects both immediate and long-term patient outcomes, even in those without prior diabetes mellitus (DM). 

Critical conditions such as AMI often present with hyperglycemia. This type of response to severe conditions occurs both in patients who suffer from DM and in those without type 2 DM, and it is linked to a poorer prognosis in both situations [1, 2]. Acute hyperglycemia can be linked to existing abnormalities in glucose metabolism or may just arise from a counterregulatory hormonal response in critical conditions. The release of stress hormones, particularly serum cortisol, catecholamines, and growth hormones, promotes gluconeogenesis and glycogenolysis. These manifestations, including neurohumoral hypersecretion and hyperglycemia, are linked to higher mortality rates in AMI, irrespective of the presence of DM [2,3].

It is a debate whether stress hyperglycemia is a direct contributor to adverse outcomes in AMI patients or simply an indicator of a more severe illness. Furthermore, individual responses to stress hyperglycemia may vary, making it challenging to generalize results and establish a prognostic impact. Despite that, many studies have demonstrated a significant correlation between blood glucose levels at admission and the severity of patient outcomes [4]. Admission hyperglycemia predicts a poor prognosis in ST-elevation myocardial infarction (STEMI) patients receiving primary percutaneous coronary intervention (PCI) [5]. A recent systemic review that utilized a comprehensive meta-analysis of 62 articles to assess various clinical outcomes in STEMI patients concluded that acute hyperglycemia has a strong correlation with negative outcomes after STEMI [6].

Although the precise mechanisms through which hyperglycemia and IR exacerbate outcomes in AMI remain largely unclear, there are several explanations for their detrimental effects. Elevated blood glucose levels can intensify myocardial damage by inducing oxidative stress and inflammation and worsening endothelial dysfunction [7]. Observations indicate elevated levels of inflammatory cytokines, including tumor necrosis factor-alpha (TNF-α) and interleukin-6 (IL-6), which further compromise insulin signaling [8-10]. Insulin resistance compromises endothelial function by diminishing the bioavailability of nitric oxide (NO). Lower levels of NO can result in increased vascular tone, which promotes vasoconstriction and elevates the risk of coronary artery blockage during an acute event. Hyperglycemia may also result in the elevated production of advanced glycation end products (AGEs), contributing to additional vascular damage and compromised myocardial function. These processes contribute to the expansion of the ischemic lesion and hinder repair mechanisms [7]. High blood glucose, along with low insulin levels and IR, both exacerbated by oxidative stress and inflammation, diminish glucose uptake and glycolysis. This results in a decreased supply of the glycolytic substrate needed for myocardial functions, leading to reliance on alternative substrates like free fatty acids. Their excessive buildup impairs contractility. Consequently, acute hyperglycemia promotes anaerobic glycolysis over oxidative phosphorylation, a shift that not only disrupts cellular energy metabolism but also heightens acidosis and intracellular calcium accumulation, thereby aggravating myocardial dysfunction and elevating the risk of arrhythmias [1, 11, 12]. Additionally, IR at the time of hospital admission for AMI is an indicator of long-term mortality, underscoring the significance of its early identification and management to enhance patient outcomes. The homeostatic model assessment of insulin resistance index (HOMA-IR), which assesses IR based on blood glucose and insulin levels, has been recently recognized as an independent predictor of in-hospital mortality for patients without type 2 DM [13-15]. Addressing stress hyperglycemia and acute IR in AMI patients may improve myocardial perfusion, diminish infarct size, and avert harmful cardiac remodeling, thus improving both immediate and long-term survival and quality of life [16].

Stress-induced hyperglycemia significantly influences neutrophil function, enhancing and impairing their activities over time. Initially, high glucose levels boost neutrophil functions that include chemotaxis, phagocytosis, radical oxygen species, and neutrophil extracellular traps (NETs) production [17, 18]. Prolonged hyperglycemia results in neutrophil dysfunction manifested by the exacerbation of inflammatory mechanisms while simultaneously diminishing reparative processes of the myocardium [19-23]. Hyperglycemic stress in AMI is associated with a prothrombotic status, as evidenced by increased platelet activation and aggregation. Neutrophils interact with platelets and endothelial cells, promoting thrombus formation in the coronary microvasculature. Enhanced neutrophil-platelet interactions and release of NETs are contributing factors to microvascular dysfunction, impaired myocardial perfusion, and adverse outcomes in patients with AMI and stress hyperglycemia [23, 24]. 

While neutrophilia is commonly observed in acute conditions, the prognostic value regarding the transient elevation of neutrophil count in cases of AMI has not been extensively researched. Researchers have found that neutrophils may indicate the severity and prognosis of AMI not only due to their accumulation, mirroring the severity of the condition via a corresponding inflammatory response, but also because their activity can lead to an adverse progression following AMI [24, 25]. Thus, after invading the necrotic area, neutrophils activate and secrete signaling molecules such as cytokines, chemokines, reactive oxygen species (ROS), and proteolytic enzymes that may result in tissue damage [26]. These activated neutrophils also release NETs, which contribute to vascular occlusion, thrombosis, and the exacerbation of myocardial injury [27].

Recent research indicates that NETs affect the size of the lesion and the remodeling of the left ventricle after an infarction [28]. Furthermore, we recently demonstrated that neutrophils undergo changes during AMI that could influence the disease's outcome [29]. Neutrophils from AMI patients with an unfavorable prognosis exhibited a marked increase in inflammatory cytokines such as CCL3, IL-1β, IL-18, and the alarmin S100A9. Additionally, our findings indicated that the p22phox and Nox2 subunits of NADPH oxidase were significantly elevated in neutrophils from patients with poor outcomes, particularly in individuals with DM [29]. This may imply that neutrophils in these patients may also contribute to increased oxidative stress within the infarcted region [30]. In turn, oxidative stress significantly contributes to the development and exacerbation of IR and hyperglycemia [31-35]. Neutrophils and stress hyperglycemia in AMI reflect a complex interplay between metabolic and immune responses.

In this study, to better comprehend the disparities between high glucose and normal to low glucose environments in the context of AMI, we focused on stress glycemia and its adverse impact on inflammation and in-hospital progression. Our examination focused on IR-specific markers in correlation with inflammatory and prognostic markers.

## Materials and methods

We conducted a longitudinal prospective study involving patients hospitalized for AMI from April 2021 to September 2022 at Elias Emergency Hospital in Bucharest, Romania. Informed consent was obtained from the participants as per the Declaration of Helsinki guidelines. The study's protocol received approval from the ethics committee of the Elias Emergency Hospital in Bucharest, Romania, under the approval number 3349/06.05.2021.

The inclusion and exclusion criteria, negative prognosis criteria, patient care, and collection and processing of blood samples were discussed in the study by Barbu et al. [29]. Specifically, patients were included within the first 24 hours of an AMI with ST-segment elevation and excluded if they had acute viral, bacterial, or fungal infections or chronic inflammatory diseases. In addition to standard blood sample collection protocols for AMI management, special probes were collected to study specific inflammatory biomarkers, including NETs, S100A8/A9, and interleukins IL-1β, IL-18, and IL-6. Given the high prevalence of stress hyperglycemia during AMI, we defined diabetic and pre-diabetic status using glycated hemoglobin (HbA1c) and medical history, in line with the American Diabetes Association (ADA) 2019 criteria [36]. For the present study, we further limited inclusion to patients who had both glycemia and HbA1c levels recorded upon admission, resulting in a total of 219 patients. Among them, 59 were non-diabetic, 78 were identified as pre-diabetic, and 82 were diabetics. The reliability of admission glucose as a marker for stress hyperglycemia has come under scrutiny [37, 38]. The stress hyperglycemia ratio (SHR) has been proposed as a more accurate index for identifying stress hyperglycemia compared to admission blood glucose, and it has shown a superior predictive value for the prognosis of AMI [39]. 

We utilized the glycemia ratio (GR) to assess the impact of stress glycemia on AMI evolution. The principle involved calculating the ratio between the blood sugar level at admission and the patient's average blood sugar, which is determined based on HbA1c. For this purpose, we used the following formula to determine GR and the following cut-offs:

$\\Calculated Average Glycaemia=HbA1\cdot35,6-77,3\\\\Glycaemia Ratio=\frac{Glycaemia (Admission)}{Calculated Average Glycaemia}$

Under-average glycemia was considered a value less than 75% of the expected glycemia value based on HbA1c. Normal glycemia was considered GR in ± 25% of the expected glycemia value based on HbA1c. Hyperglycemia, or stress hyperglycemia, was considered more than 125% of the expected glycemia value based on HbA1c. After applying these conditions, the batch was divided into three groups for comparison. The parameters under study were then assessed for correlations and prognostic implications. A negative prognosis or a complicated in-hospital course was defined by the presence of any of the following five conditions: development of Killip class 3 or 4, left ventricular ejection fraction (LVEF) at discharge less than 40%, complex ventricular arrhythmias, mechanical complications, or death.

The HOMA index, an independent predictor of in-hospital mortality that evaluates IR and pancreatic beta cell function, was calculated for each patient using the following formula:

\begin{equation*} \text{HOMA-IR} = \frac{\text{insulin} \ (\mu\text{U/mL}) \times \text{blood glucose} \ (\text{mg/dL})}{405} \label{eq:homa_ir} \end{equation*}

Data processing, statistical analysis, and visualizations were performed using Python. Group differences were assessed with Tukey's Honestly Significant Difference (HSD) test, and correlations were tested using the Pearson R coefficient. The analyses were conducted using functions from the statsmodels and scipy packages in Python 3 (Python Software Foundation, Fredericksburg, VA). Graphs were plotted with the Matplotlib (The Matplotlib development team) and Seaborn (Waskom, ML, (2021)) libraries.

## Results

Clinical data and mean parameter values for the groups formed after applying the formula are summarized in Table 1.

**Table 1:** Summary of patient demographics and paraclinical markers in the study population after applying the formulas *Data are reported as median (quantile 5%—quantile 95%); ** Data are reported as a fraction (percent), where differences between the fraction denominator and the number of patients in the corresponding group indicate missing values in the data. F: female; M: male; ESR: erythrocyte sedimentation rate; CRP: C reactive protein; IL: interleukin; LVEF: left ventricle ejection fraction; WBC: white blood cells; NLR: neutrophil-to-lymphocyte ratio; NETs: neutrophil extracellular traps

	Under-average glycemia	Normal glycemia ratio	Stress glycemia ratio
Number	40	149	30
Sex			
F	13 (32.5%)	50 (33.6%)	10 (33.3%)
M	27 (67.5%)	99 (66.4%)	20 (66.6%)
Diagnosis			
Non-diabetic (N)	3 (7.5%)	47 (31.5%)	9 (30.0%)
Pre-diabetic (PD)	18 (45.0%)	57 (38.3%)	3 (10.0%)
Diabetic (D)	19 (47.5%)	45 (30.2%)	18 (60.0%)
Prognosis			
Favorable	29 (72.5%)	104 (69.8%)	13 (43.3%)
Unfavorable	11 (27.5%)	45 (30.2%)	17 (56.7%)
Age (years)	59.60 (41.68 - 74.91)	60.30 (40.62 - 80.62)	68.05 (53.43 - 84.34)
Troponin (pg/ml)	37.5 (2.5 - 80)	35.85 (0.71 - 129)	32.60 (2.26 - 80)
D-Dimer (microg/ml)	187 (46.2 - 1419)	194.5 (63.3 - 863.8)	400 (117.4 - 1319.4)
ESR (mm/h)	15 (6 - 75.8)	18.00 (5.00 - 61.00)	18.00 (5.75 - 85.5)
CRP (admission) (mg/dl)	10.00 (5.00 - 253.48)	8.35 (5 - 100)	8.60 (4.21 - 125.73)
Fibrinogen (mg/dl)	391 (242.8 - 806.4)	381.5 (246.1 - 559.3)	373 (238- 687)
Gas6 (ng/ml)	11.84 (6 - 32.95)	11.97 (6.69 - 24.84)	11.93 (7.62 - 31.74)
Glycemia (admission) (mg/dl)	102 (72.5 - 195.15)	124 (93.4 - 294.8)	215 (149.05 - 355.80)
IL-18 (pg/m)	181 (110.5 - 728.3)	169.2 (114.6 - 519.6)	189.3 (118.3 - 1228.8)
IL-1b (pg/ml)	1.70 (0.00 - 37.90)	2.13 (0 - 47.51)	2.02 (0.35 - 160.34)
IL-6 (pg/ml)	25.57 (6.83 - 291.77)	18.52 (5.29 - 96.84)	28.48 (10.36 - 64.37)
Insulinemia( microIU/ml)	14.46 (3.05 - 32.31)	12.84 (3.53 - 44.00)	11.41 (5.24 - 30.39)
LVEF (Admission) (%)	45.00 (30.00 - 55.00)	45.00 (30.00 - 55.00)	40.00 (24.00 - 50.00)
WBC (*1000microL)	11.86 (7.31 - 16.40)	12.32 (7.30 - 18.18)	13.57 (7.89 - 22.65)
Lymphocytes (*1000microL)	1.93 (0.74 - 3.92)	1.57 (0.70 - 3.59)	1.32 (0.37 - 3.27)
NLR	3.89 (1.75 - 9.95)	5.31 (2.32 - 14.65)	10.36 (2.76 - 39.37)
Neutrophils (**1000microL)	8.54 (5.07 - 12.04)	9.40 (4.70 - 15.12)	11.10 (5.23 - 20.67)
S100A8/9 (ng/ml)	3008 (619.4 - 5556.2)	2078.7 (467.7 - 6205.8)	2642.4 (998.2 - 6231.8)
NETs (Sytox Green)	178.9 (64.6 - 430.1)	117.9 (54.2 - 487.9)	157.5 (85.7 - 421.4)

The majority of patients presented with a normal glycemia ratio upon admission. Among those with stress-induced hyperglycemia, diabetics constituted 60%, non-diabetics 30%, and prediabetics 10%.

**Figure 1:**
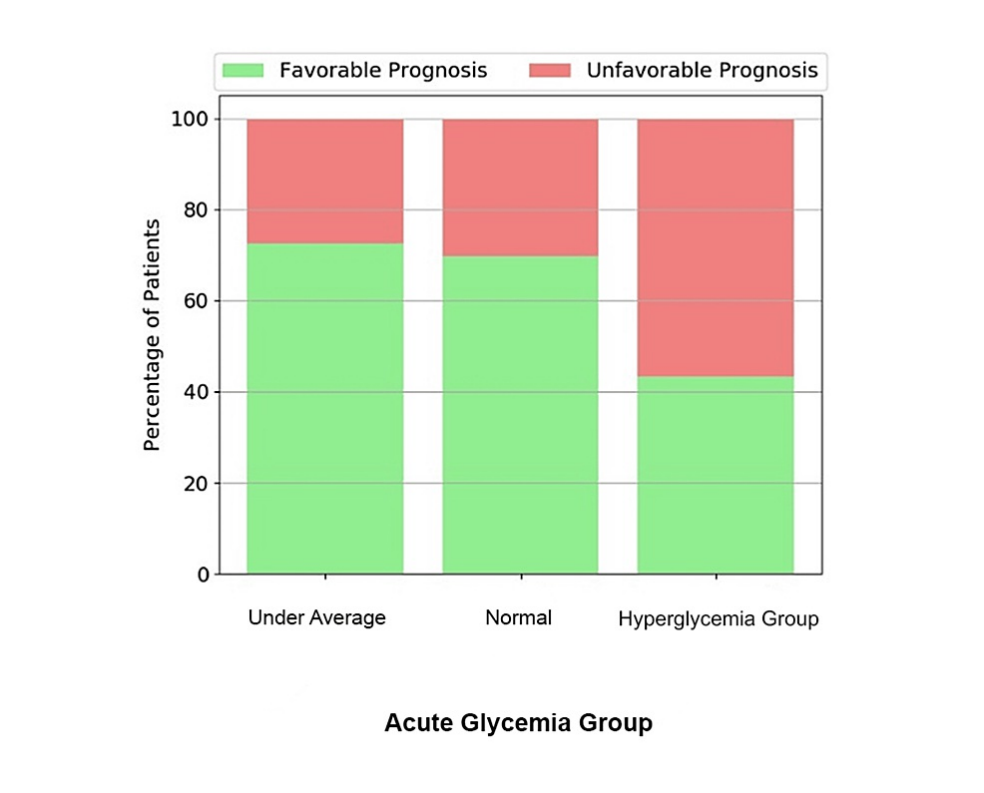
Prognosis depending on acute glycemia group: under average, normal, and hyperglycemia

The majority of patients in the stress hyperglycemia group (17/30, or 56.7%) had unfavorable prognoses (Figure 1).

The best prognosis was registered in the under-average glycemia group. Although under-average for each individual patient, absolute blood sugar values in this group were between 72.5 and 195.15 mg/dl, with a median of 102. There were no values consistent with hypoglycemia in this group.

**Figure 2:**
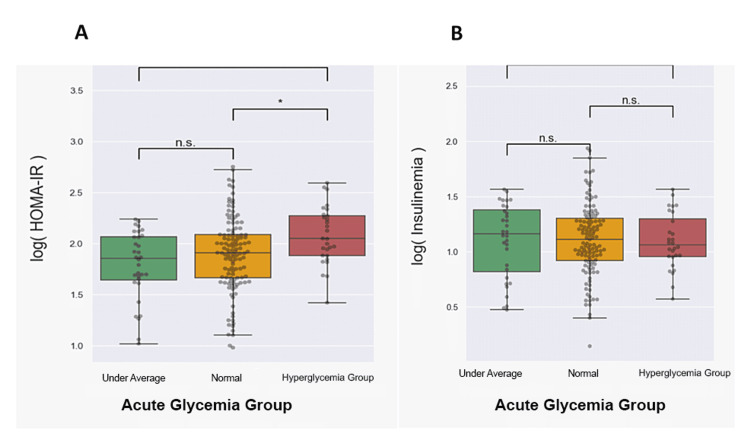
Comparison between HOMA-IR values and insulinemia values in acute glycemia groups (A) HOMA-IR values between acute glycemia groups; (B) insulinemia values between acute glycemia groups. The statistical significance shown on the graph refers to the outcomes of Tukey's test for multiple comparisons. (n.s., not significant; *p < 0.05; **p < 0.01) HOMA-IR: homeostatic model assessment of insulin resistance index

Typically, the HOMA-IR index is statistically higher in patients with elevated blood sugar levels (Figure 2A). Insulinemia exhibits the highest values in the under-average group and the lowest in the stress hyperglycemia group, yet the differences are not statistically significant (Figure 2B). In our study cohort, the HOMA-IR index is mostly influenced by glucose levels.

**Figure 3:**
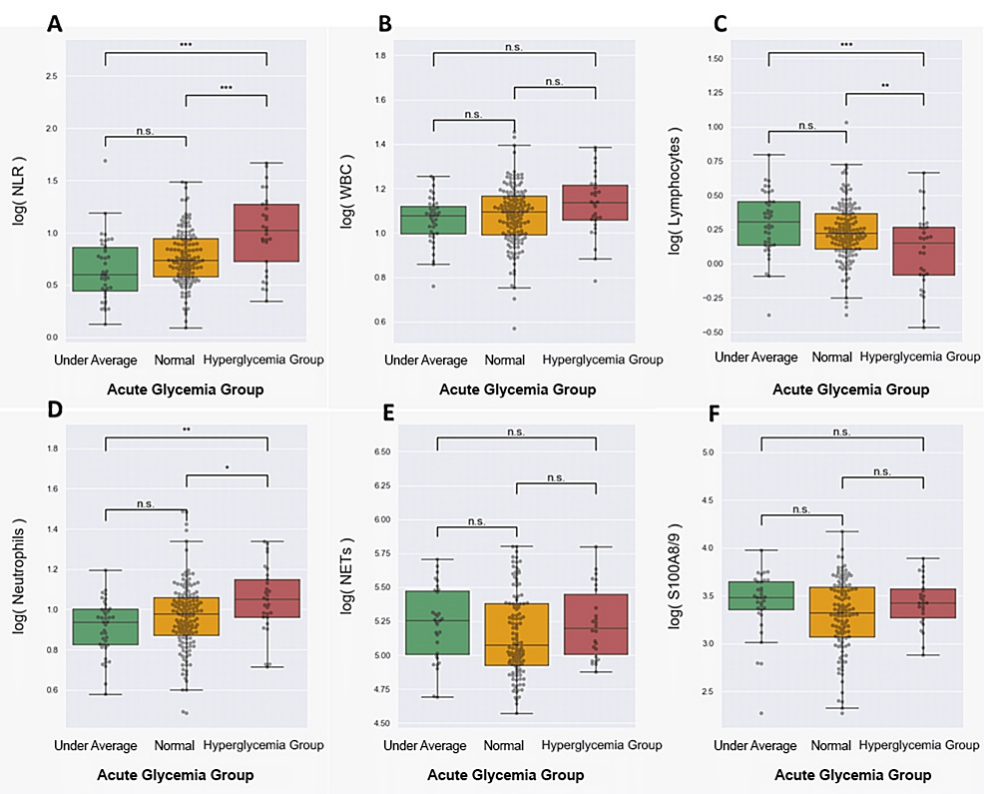
Parameters values between acute glycemia groups (A) neutrophil-to-lymphocyte ratio (NLR); (B) white blood cells (WBC); (C) lymphocytes; (D) neutrophils; (E) neutrophil extracellular traps (NETs); (F) S100A8/A9. The statistical significance shown on the graph refers to the outcomes of Tukey's test for multiple comparisons (n.s.: not significant, *p < 0.05, **p < 0.01).

The neutrophil-to-lymphocyte ratios (NLR) (Figure 3A) and neutrophils (Figure 3D) had significantly higher values in the stress glycemia group. As expected, lymphocytes registered decreased values in this group (Figure 3C). White blood cells (Figure 3B) and neutrophil activity parameters such as NETs (Figure 3E) and S100A8/A9 (Figure 3F) did not differ between glycemia groups in the first 24 hours after AMI.

**Figure 4:**
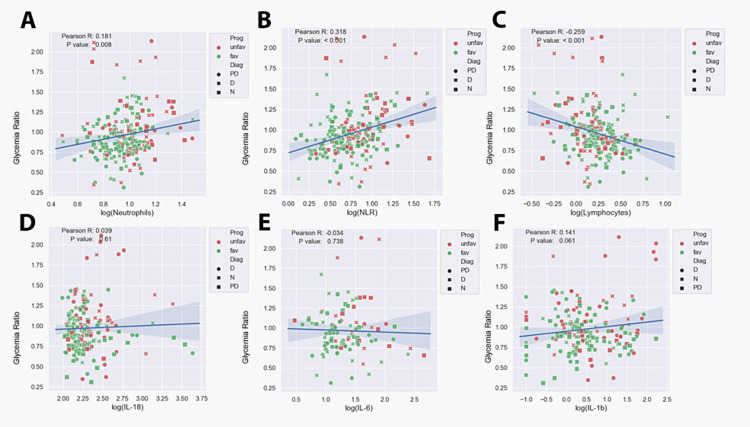
A scatterplot showing the correlation between glycemia ratio and the following inflammatory markers: (A) neutrophils, (B) neutrophil-to-lymphocyte ratio (NLR), (C) lymphocytes, (D) interleukin (IL)-18, (E) IL-6, and (F) IL-1 beta. N: non-diabetic; PD: pre-diabetic; D: diabetic; prog: prognosis; fav: favorable; unfav: unfavorable

**Figure 5:**
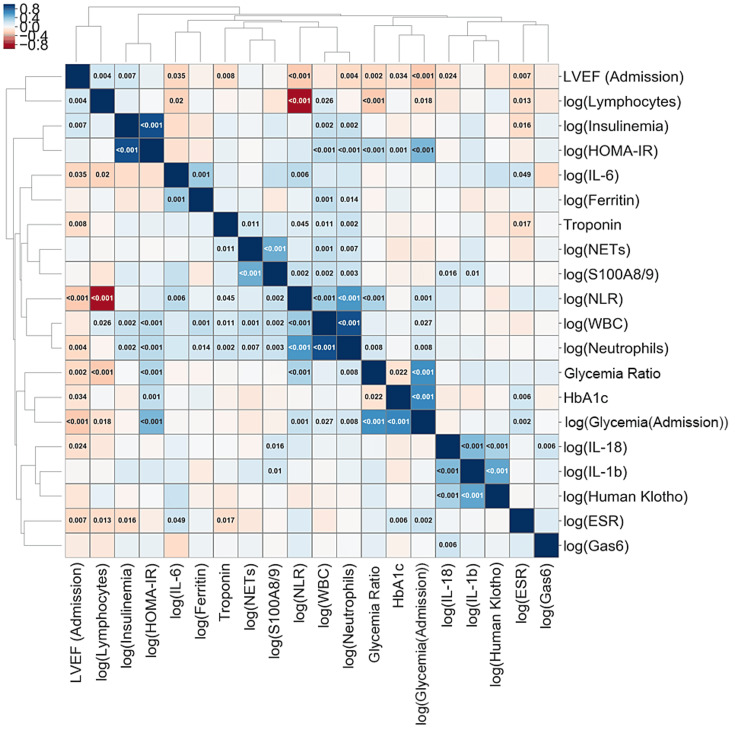
Correlation matrix for inflammatory and glycemic markers Cell colors indicate the direction and strength of correlations: blue (for positive correlations), red (for negative correlations), and orange (for significant correlations). The values indicated in the cells represent the p-value of the correlation. The parameters are grouped according to their similarity of association with the other parameters by the unweighted pair group method with an arithmetic mean. LVEF: left ventricular ejection fraction; HOMA-IR: homeostatic model assessment of insulin resistance; IL: interleukin; NETs: neutrophil extracellular traps; NLR: neutrophil-to-lymphocyte ratio; WBC: white blood cell; HbA1C: glycated hemoglobin; ESR: erythrocyte sedimentation rate

The GR was significantly and positively correlated with certain inflammation markers, such as neutrophil count (Pearson R = 0.181, p = 0.008) (Figure 4A) and parameters derived from neutrophils, like the NLR (Pearson R = 0.318, p<0.001) (Figure 4B). As expected, blood glucose levels were significantly and negatively correlated with lymphocyte counts (Pearson R = -0.259, p<0.001). Interestingly, the glycemia ratio did not correlate with other evaluated inflammatory markers (Figures 4D-4F, Figure 5). Additionally, a significant negative correlation was found between GR and LVEF at admission (p = 0.002) (Figure 5). The acute insulin resistance index, known as the HOMA-IR index, is also positively correlated with neutrophils, WBC, and NLR, with a p-value of less than 0.001 for all three parameters. There was a negative correlation between LVEF and GR (p = 0.002) (Figure 5). No significant correlations were observed between the neutrophil response and HBA1c levels, indicating that chronic blood glucose levels may not significantly influence the acute neutrophil response (Figure 5).

## Discussion

In daily practice, clinicians can indirectly assess the body's response to critical injuries and acute stress, such as AMI, through cost-effective, standard blood tests. These tests include white blood cell count, neutrophil count, NLR, C-reactive protein (CRP) levels, blood sugar, hepatic enzymes, and lactate dehydrogenase [27, 30, 35, 40]. These biomarkers are elevated in clinical observations, widely confirmed by studies, and may predict adverse outcomes. Stress hyperglycemia, characterized by elevated blood glucose levels in response to stress, is frequently seen in patients suffering from AMI. It is associated with the body's immediate stress reaction and carries important consequences for patient outcomes. Insulin administration during the acute phase of AMI has been shown to improve survival, suggesting that hyperglycemia is more than just an epiphenomenon of stress but may also be a modifiable risk factor in AMI [7, 41-44].

Existing data have already linked acute hyperglycemia with increased mortality and morbidity in AMI, independent of DM status [2, 3]. In our study, unfavorable evolution was also more frequent in the acute stress hyperglycemia group. The observation that 60% of the patients in the stress hyperglycemia group had DM could have impacted this outcome, as DM was proven to be an independent factor for adverse outcomes in this study cohort [29]. Yet, in non-diabetic patients experiencing STEMI, research indicates that stress hyperglycemia and acute IR correlate with incomplete myocardial reperfusion and compromised coronary microcirculation after PCI [45]. Such impaired reperfusion is associated with a higher rate of adverse outcomes, including increased infarct size and more severe left ventricular remodeling. Moreover, acute IR may cause left ventricular remodeling, worsening of ejection fraction, and enlarged ventricular volumes, which predict a worse prognosis and elevated mortality rates independent of DM status [9, 14, 45]. Additionally, our findings indicate that the glycemia ratio also negatively correlates with the LVEF.

It is noteworthy that the most favorable prognosis occurred in the group with blood sugar levels below average. Notably, there were no instances of hypoglycemia, as values did not fall below 70 mg/dl in this group. The data indicate that lower-than-average blood sugar levels may lead to a better prognosis.

Insulinemia exhibits the highest values in the under-average group and the lowest in the stress hyperglycemia group, suggesting that low insulin secretion may explain high blood sugar levels in the acute stress hyperglycemia ratio group rather than IR. Yet, insulinemia levels are not statistically different between groups.

Growing evidence underscores the significance of the innate immune response in the development of acute coronary syndrome, and recent studies indicate a modified immune cell phenotype, including neutrophils, in this scenario [46, 47]. Drawn to the infarcted area by cellular debris and inflammatory signals from necrotic cells, neutrophils quickly converge on the ischemic region to begin the inflammatory response. They generate and secrete signaling molecules such as cytokines, chemokines, ROS, and proteolytic enzymes that may result in tissue damage [26]. These activated neutrophils also release NETs, which contribute to vascular occlusion, thrombosis, and the exacerbation of myocardial injury [27]. Recent research indicates that NETs affect the size of the lesion and the remodeling of the left ventricle after an infarction. Moreover, an increased NLR is correlated with a poor prognosis in AMI. Dynamic shifts in NLR can precede clinical manifestations by hours, offering prompt prognostic insights [48]. The quantity and activity of neutrophils have been shown to affect the short-term prognosis of patients with myocardial infarction, as we previously showed [29]. The NLR can predict both short-term and long-term mortality, demonstrating a strong correlation with the Global Registry of Acute Coronary Events (GRACE) and Synergy Between Percutaneous Coronary Intervention With Taxus and Cardiac Surgery (SYNTAX) scores [49]. The notable differences in the inflammatory profiles of neutrophils among AMI patients with adverse in-hospital outcomes point to potential changes in the innate immune response that could negatively impact disease progression [29]. 

Stress hyperglycemia has been shown to intensify pro-inflammatory processes and adversely affect the innate immune system [7]. In the hyperglycemic environment, there is an increased expression of adhesion molecules for neutrophils and an increased release of cytokines, chemokines, and ROS. Oxidative stress activates neutrophils and promotes the release of pro-inflammatory cytokines like TNF-α and IL-6, which exacerbate IR by impairing insulin signaling. Reactive oxygen species can directly oxidize insulin receptors and downstream signaling molecules, affecting insulin-stimulated glucose uptake in target tissues [31-35]. Activated neutrophils can constitute a source of ROS in a hyperglycemic environment, as we demonstrated previously [29]. The consequence is an inflammatory loop perpetuated by the strong interplay between metabolic and inflammatory disturbances.

In this study, the findings indicate that acute hyperglycemia correlates with inflammation in AMI, as seen with neutrophil count and NLR. The catecholaminergic response to a critical illness may elevate both blood glucose and systemic inflammatory markers. Also, neutrophils are the first host defense cells that respond to injury. Following a myocardial infarction, neutrophils are rapidly recruited to the ischemic area, where they begin the inflammatory response. Yet, blood glucose did not correlate with other inflammation parameters within the first 24 hours post-symptom onset. Other mechanisms on top of stress hormone activity may explain the connection between stress hyperglycemia and high neutrophil values. Acutely elevated glucose levels and IR are known to increase the expression of adhesion molecules on neutrophils and endothelial cells, aiding their migration to the inflamed myocardium [20, 49]. Chronic glycemic status, as measured by HbA1c, showed no correlation with neutrophil activity.

Our research has identified robust correlations between markers of acute IR and neutrophil activity. However, it is crucial to acknowledge that correlation does not imply causation. To definitively establish a causal link between stress-induced glycemia, neutrophil activity, and the underlying pathological mechanisms, further research and comprehensive clinical trials are essential.

While our study provides valuable insights into the relationship between stress hyperglycemia, IR, and inflammation in AMI patients, several limitations should be noted. The single-center design and sample size of 219 patients may limit the generalizability of our findings. The study's focus on immediate outcomes without long-term follow-up restricts our understanding of persistent effects. Potential confounding factors, such as comorbidities and medications, may not have been fully accounted for. Additionally, the reliance on the GR and HOMA-IR index might not capture the complete metabolic complexity of critical illness, and our selective focus on certain inflammatory markers could overlook other important pathways. Finally, our study population from Bucharest, Romania, may not reflect broader ethnic and genetic diversity. Future research addressing these limitations will be essential for a comprehensive understanding of the metabolic and inflammatory dynamics of AMI.

## Conclusions

Overall, our data present evidence that the poorer in-hospital evolution of AMI patients may be related to stress hyperglycemia measured by GR. The link between stress hyperglycemia, IR, and negative cardiac remodeling highlights the necessity for meticulous metabolic management in AMI patients. Acute IR, defined by GR and HOMA-IR index, is strongly correlated with neutrophil count and NLR within the first 24 hours after AMI onset and is not correlated with other inflammatory molecules such as interleukins IL-1β, IL-18, and IL-6. In conclusion, standard blood tests can provide valuable insights into the AMI prognosis. Stress hyperglycemia, frequently observed in AMI patients, is linked to poorer outcomes and may serve as a modifiable risk factor. There is a complex interplay between metabolic and immune responses in AMI, as seen with the correlation between stress hyperglycemia and inflammation, particularly neutrophil activity.

## References

[1] Chakrabarti AK, Singh P, Gopalakrishnan L (2012). Admission hyperglycemia and acute myocardial infarction: outcomes and potential therapies for diabetics and nondiabetics. Cardiol Res Pract.

[2] Swieszkowski SP, Costa D, Aladio JM (2022). Neurohumoral response and stress hyperglycemia in myocardial infarction. J Diabetes Complications.

[3] Capes SE, Hunt D, Malmberg K, Gerstein HC (2000). Stress hyperglycaemia and increased risk of death after myocardial infarction in patients with and without diabetes: a systematic overview. Lancet.

[4] Falciglia M, Freyberg RW, Almenoff PL, D'Alessio DA, Render ML (2009). Hyperglycemia-related mortality in critically ill patients varies with admission diagnosis. Crit Care Med.

[5] Chen PC, Chua SK, Hung HF (2014). Admission hyperglycemia predicts poorer short- and long-term outcomes after primary percutaneous coronary intervention for ST-elevation myocardial infarction. J Diabetes Investig.

[6] Alkatiri AH, Qalby N, Mappangara I, Zainal AT, Cramer MJ, Doevendans PA, Qanitha A (2024). Stress hyperglycemia and poor outcomes in patients with ST-elevation myocardial infarction: a systematic review and meta-analysis. Front Cardiovasc Med.

[7] Marfella R, Siniscalchi M, Esposito K (2003). Effects of stress hyperglycemia on acute myocardial infarction: role of inflammatory immune process in functional cardiac outcome. Diabetes Care.

[8] Shoelson SE, Lee J, Goldfine AB (2006). Inflammation and insulin resistance. J Clin Invest.

[9] Yang CD, Shen Y, Lu L (2019). Insulin resistance and dysglycemia are associated with left ventricular remodeling after myocardial infarction in non-diabetic patients. Cardiovasc Diabetol.

[10] Brooks-Worrell B, Palmer JP (2012). Immunology in the Clinic Review Series; focus on metabolic diseases: development of islet autoimmune disease in type 2 diabetes patients: potential sequelae of chronic inflammation. Clin Exp Immunol.

[11] Incalza MA, D'Oria R, Natalicchio A, Perrini S, Laviola L, Giorgino F (2018). Oxidative stress and reactive oxygen species in endothelial dysfunction associated with cardiovascular and metabolic diseases. Vascul Pharmacol.

[12] Dhalla NS, Temsah RM, Netticadan T (2000). Role of oxidative stress in cardiovascular diseases. J Hypertens.

[13] Moura FA, Carvalho LS, Cintra RM (2014). Validation of surrogate indexes of insulin sensitivity in acute phase of myocardial infarction based on euglycemic-hyperinsulinemic clamp. Am J Physiol Endocrinol Metab.

[14] Al-Ali SA, Alidrisi HA, Hameed A (2022). Correlation of insulin resistance with short-term outcome in nondiabetic patients with ST-segment elevation myocardial infarction. Cureus.

[15] Trifunovic D, Stankovic S, Sobic-Saranovic D (2014). Acute insulin resistance in ST-segment elevation myocardial infarction in non-diabetic patients is associated with incomplete myocardial reperfusion and impaired coronary microcirculatory function. Cardiovasc Diabetol.

[16] Tenerz A, Norhammar A, Silveira A, Hamsten A, Nilsson G, Rydén L, Malmberg K (2003). Diabetes, insulin resistance, and the metabolic syndrome in patients with acute myocardial infarction without previously known diabetes. Diabetes Care.

[17] Uribe-Querol E, Rosales C (2022). Neutrophils actively contribute to obesity-associated inflammation and pathological complications. Cells.

[18] Feng X, Yu F, Wei M (2023). The association between neutrophil counts and neutrophil-to-lymphocyte ratio and stress hyperglycemia in patients with acute ischemic stroke according to stroke etiology. Front Endocrinol (Lausanne).

[19] Shafqat A, Abdul Rab S, Ammar O (2022). Emerging role of neutrophil extracellular traps in the complications of diabetes mellitus. Front Med (Lausanne).

[20] Thimmappa PY, Vasishta S, Ganesh K, Nair AS, Joshi MB (2023). Neutrophil (dys)function due to altered immuno-metabolic axis in type 2 diabetes: implications in combating infections. Hum Cell.

[21] Dowey R, Iqbal A, Heller SR, Sabroe I, Prince LR (2021). A bittersweet response to infection in diabetes; targeting neutrophils to modify inflammation and improve host immunity. Front Immunol.

[22] Scherer AK, Hopke A, Sykes DB, Irimia D, Mansour MK (2021). Host defense against fungal pathogens: adaptable neutrophil responses and the promise of therapeutic opportunities?. PLoS Pathog.

[23] Döring Y, Libby P, Soehnlein O (2020). Neutrophil extracellular traps participate in cardiovascular diseases: recent experimental and clinical insights. Circ Res.

[24] Ma Y (2021). Role of neutrophils in cardiac injury and repair following myocardial infarction. Cells.

[25] Tian J, Liu Y, Liu Y (2017). Prognostic association of circulating neutrophil count with no-reflow in patients with ST-segment elevation myocardial infarction following successful primary percutaneous intervention. Dis Markers.

[26] Selders GS, Fetz AE, Radic MZ, Bowlin GL (2017). An overview of the role of neutrophils in innate immunity, inflammation and host-biomaterial integration. Regen Biomater.

[27] Döring Y, Soehnlein O, Weber C (2017). Neutrophil extracellular traps in atherosclerosis and atherothrombosis. Circ Res.

[28] Li T, Yan Z, Fan Y, Fan X, Li A, Qi Z, Zhang J (2022). Cardiac repair after myocardial infarction: a two-sided role of inflammation-mediated. Front Cardiovasc Med.

[29] Barbu E, Mihaila AC, Gan AM (2024). The elevated inflammatory status of neutrophils is related to in-hospital complications in patients with acute coronary syndrome and has important prognosis value for diabetic patients. Int J Mol Sci.

[30] Thorand B, Kolb H, Baumert J (2005). Elevated levels of interleukin-18 predict the development of type 2 diabetes: results from the MONICA/KORA Augsburg Study, 1984-2002. Diabetes.

[31] Hotamisligil GS (2006). Inflammation and metabolic disorders. Nature.

[32] Rolo AP, Palmeira CM (2006). Diabetes and mitochondrial function: role of hyperglycemia and oxidative stress. Toxicol Appl Pharmacol.

[33] Rains JL, Jain SK (2011). Oxidative stress, insulin signaling, and diabetes. Free Radic Biol Med.

[34] Gerber PA, Rutter GA (2017). The role of oxidative stress and hypoxia in pancreatic beta-cell dysfunction in diabetes mellitus. Antioxid Redox Signal.

[35] Newsholme P, Keane KN, Carlessi R, Cruzat V (2019). Oxidative stress pathways in pancreatic β-cells and insulin-sensitive cells and tissues: importance to cell metabolism, function, and dysfunction. Am J Physiol Cell Physiol.

[36] American Diabetes Association (2020). 2. Classification and diagnosis of diabetes: standards of medical care in diabetes-2020. Diabetes Care.

[37] Ishihara M, Kagawa E, Inoue I (2007). Impact of admission hyperglycemia and diabetes mellitus on short- and long-term mortality after acute myocardial infarction in the coronary intervention era. Am J Cardiol.

[38] Schmitz T, Freuer D, Harmel E, Heier M, Peters A, Linseisen J, Meisinger C (2022). Prognostic value of stress hyperglycemia ratio on short- and long-term mortality after acute myocardial infarction. Acta Diabetol.

[39] Xu W, Yang YM, Zhu J, Wu S, Wang J, Zhang H, Shao XH (2022). Predictive value of the stress hyperglycemia ratio in patients with acute ST-segment elevation myocardial infarction: insights from a multi-center observational study. Cardiovasc Diabetol.

[40] Karadeniz M, Duran M, Akyel A (2015). High sensitive CRP level is associated with intermediate and high syntax score in patients with acute coronary syndrome. Int Heart J.

[41] Malmberg K, Rydén L, Wedel H (2005). Intense metabolic control by means of insulin in patients with diabetes mellitus and acute myocardial infarction (DIGAMI 2): effects on mortality and morbidity. Eur Heart J.

[42] Malmberg K, Norhammar A, Wedel H, Rydén L (1999). Glycometabolic state at admission: important risk marker of mortality in conventionally treated patients with diabetes mellitus and acute myocardial infarction: long-term results from the Diabetes and Insulin-Glucose Infusion in Acute Myocardial Infarction (DIGAMI) study. Circulation.

[43] Ritsinger V, Malmberg K, Mårtensson A (2014). Intensified insulin-based glycaemic control after myocardial infarction: mortality during 20 year follow-up of the randomised Diabetes Mellitus Insulin Glucose Infusion in Acute Myocardial Infarction (DIGAMI 1) trial. Lancet Diabetes Endocrinol.

[44] Malmberg K, Rydén L, Efendic S (1995). Randomized trial of insulin-glucose infusion followed by subcutaneous insulin treatment in diabetic patients with acute myocardial infarction (DIGAMI study): effects on mortality at 1 year. J Am Coll Cardiol.

[45] Kasem SM, Saied GM, Hegazy AN, Abdelsabour M (2021). Impact of acute insulin resistance on myocardial blush in non-diabetic patients undergoing primary percutaneous coronary intervention. Front Cardiovasc Med.

[46] Maréchal P, Tridetti J, Nguyen ML (2020). Neutrophil phenotypes in coronary artery disease. J Clin Med.

[47] Biasucci LM, Liuzzo G, Grillo RL (1999). Elevated levels of C-reactive protein at discharge in patients with unstable angina predict recurrent instability. Circulation.

[48] Tavares F, Moraes PI, Souza JM (2022). Prognostic role of neutrophil-to-lymphocyte ratio in patients with ST-elevation myocardial infarction undergoing to pharmaco-invasive strategy. Cardiovasc Revasc Med.

[49] Kurup R, Patel S (2017). Neutrophils in acute coronary syndrome. EMJ Cardiol Cardiol.

